# Signal combination in flutter vibration perception

**DOI:** 10.1371/journal.pone.0350140

**Published:** 2026-06-11

**Authors:** Shasha Wei, Alex R. Wade, Catherine E.J. Preston, Daniel H. Baker

**Affiliations:** 1 Department of Psychology, University of York, York, United Kingdom; 2 York Biomedical Research Institute, University of York, York, United Kingdom; University of Giessen: Justus-Liebig-Universitat Giessen, GERMANY

## Abstract

While the brain’s integration of auditory and visual inputs has been extensively investigated, the mechanisms underlying somatosensory signal combination remain less explored. Here, we combine psychophysical thresholds with steady-state somatosensory evoked potentials (SSSEPs) to investigate how vibrotactile inputs are combined across fingers. We find that doubling the number of stimulated digits leads to a weak improvement in detection threshold, consistent with probability summation, whereas introducing a masking stimulus to interleaved digits induces inter-digit suppression. Correspondingly, EEG recordings reveal a ~ 1.4-fold increase in SSSEP amplitude when doubling the number of digits stimulated at the same frequency, reflecting a summation effect. In contrast, SSSEP amplitudes decrease when digits are vibrated at two different frequencies, further supporting the presence of suppression. These results are consistent with a model featuring inhibition between digits and reveal that the weight of suppression is intermediate between that observed in binocular vision and binaural hearing.

## Introduction

Signal combination is crucial for human perception and our interaction with the environment. Our sensory systems comprise a variety of receptors and neural pathways that detect and process a wide range of stimuli, including sight, hearing, touch, and smell. To construct a coherent and comprehensive representation of our surroundings and our own body, the brain must filter out overlapping or redundant information and avoid excessively strong signals to prevent sensory overload. Therefore, in addition to additive processes, suppressive processes are also involved in signal combination. This suppression during signal combination has been investigated both within (e.g., vision [1], hearing [2], touch [3,4]) and between (e.g., visuo-tactile [5], audio-visual [6]) modalities.

**Fig 1:**
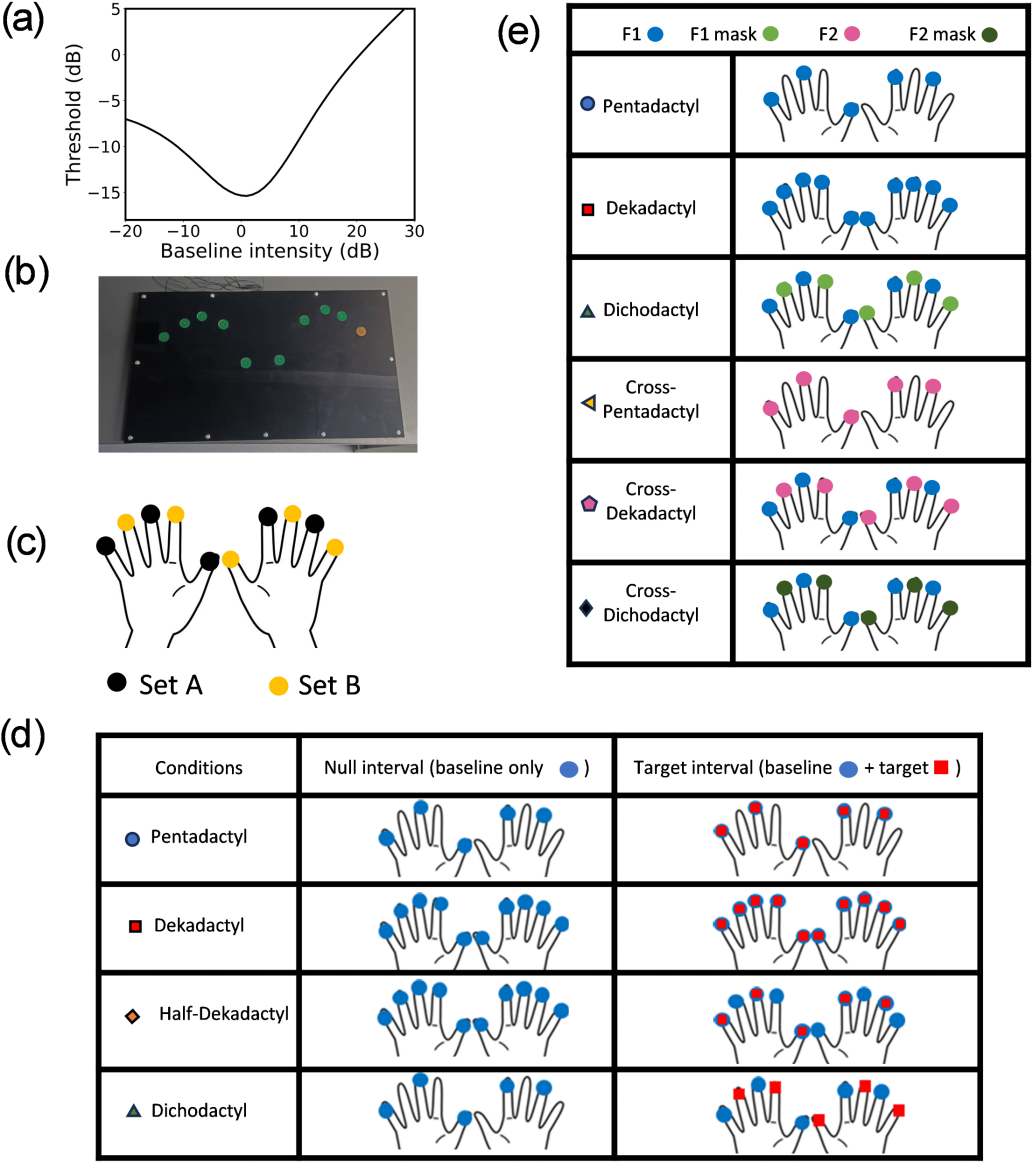
Overview of methods details. Panel (a) shows an example ‘dipper’ function. Panel (b) shows the board containing the ten tactors. Panel (c) illustrates the allocation of digits into two sets. Panel (d) illustrates the arrangements of baseline (blue circles) and target (red squares) stimuli in the two intervals of a trial in Experiment 1, for four stimulus arrangements (rows). Panel (e) illustrates the stimulus conditions used in Experiment 2. F1 (26 Hz) and F2 (23 Hz) indicate the flutter stimulus frequencies. F1 mask and F2 mask refer to flutter stimuli presented at the same respective frequencies with a fixed intensity of 32% of the maximum.

In visual and auditory perception, psychophysical studies have shown that detection performance for binocular or binaural presentation is typically between a factor of √2 and 2 better than for monocular or monaural presentation [2,7,8]. This summation at threshold implies the existence of physiological mechanisms that combine signals across eyes or ears. Above threshold, detection performance improves when a weak fixed intensity (‘baseline’) stimulus is added; a phenomenon known as facilitation. At higher baseline intensities performance worsens, producing a masking effect. When plotted against baseline intensity, the discrimination thresholds exhibit a “dipper” shape (see Fig 1a). The facilitation and masking effects arise from the brain transducing physical signals into neural responses in a non-linear manner, and are observed for many sensory stimuli (see ref. [9]).

To complement psychophysical work, we can directly measure the response to stimuli of different intensities by recording brain activity. One convenient method is the steady state technique, in which periodic stimulus oscillations are reflected in electromagnetic neural responses at the same frequency, which can be recorded using EEG or MEG [10,11]. For instance, some EEG studies have investigated the signal combination process in visual and auditory modalities by recording steady-state signals [1,2]. These studies found that responses increased when input channels (eyes or ears) were doubled, but by less than a factor of two. Additionally, when a mask was added to one channel instead of a signal input (i.e., oscillating at a different frequency), the signal response decreased due to suppression [12].

The process by which the brain combines multiple signals has attracted great interest in recent years, leading to the development of several computational models. The two stage gain control model of signal combination [13] successfully accounts for both binocular and binaural perception, positing that the signal from one channel (left or right) is inhibited by the other channel before being summed. The equations describing this model are defined as:


$$\begin{array}{c} {Stage\text{1}}_{L}\;=\;\frac{\overset{m}{\underset{L}{I}}}{S+I_{L}+\omega I_{R}}\; \end{array}$$ (1)

$$\begin{array}{c} {Stage\text{1}}_{R}\;=\;\frac{\overset{m}{\underset{R}{I}}}{S+I_{R}+\omega I_{L}}\; \end{array}$$ (2)

$$\begin{array}{c} sum=\;{Stage\text{1}}_{L}+\;{Stage\text{1}}_{R} \end{array}$$ (3)

$$\begin{array}{c} esp=\;\frac{{sum}^{p}}{Z\;+\;{sum}^{q}} \end{array}$$ (4)

In this model, sensory inputs from adjacent channels are processed in two stages. At the first stage, each input undergoes gain control, where the response from one channel is suppressed by the input from the other channel. This is described by Equations 1 and 2. For example, the left channel response (Stage1_*L*_) depends not only on its own stimulus intensity (I_*L*_) but also on the intensity of the right channel (I_*R*_), modulated by the suppression weight 𝜔. The exponent 𝑚 and saturation constant *S* are free parameters and determine how the input is transformed nonlinearly. The outputs of the left and right channels are then summed to form 𝑠𝑢𝑚 (Equation 3), which represents the combined signal strength after inter-channel suppression. This combined signal is then passed through a nonlinear transducer function (Equation 4), which produces the final response (𝑟e𝑠𝑝) based on the free parameters 𝑝, 𝑞 and 𝑍. These parameters govern the gain and saturation characteristics of the system. In vision, the weight of suppression is approximately 𝜔 = 1, whereas in auditory perception, suppression between the ears is dramatically weaker, with 𝜔 close to 0. Therefore, while suppression can be key to signal combination, its strength varies across different sensory modalities.

In tactile perception, neuroimaging findings also suggest the presence of summation and suppression of vibrotactile signals. For instance, studies have recorded brain responses to vibrotactile stimuli delivered to two fingers separately and simultaneously. Results indicated that at low levels of stimulation near the perceptual threshold, there was a facilitation or summation effect between two fingers. However, at higher levels of stimulation, a suppression effect was observed [4]. Furthermore, the brain’s response to simultaneous tactile stimulation of two fingers is less than (approximately 50% of) the sum of the responses to individual finger stimuli [3,4]. In addition, the extent of suppression is dependent on the spatial distance between the fingers [4,14]. In some psychophysical studies, researchers measured vibrotactile stimulus detection thresholds using various contactor sizes [15,16]. Results showed that the threshold decreased as the contactor size increased, a phenomenon known as area summation. Specifically, when the contactor size was doubled, the threshold decreased by approximately 3 dB (a factor of 1.4) [15]. These findings focused on the summation rather than suppression between inputs, because suppressive processes are minimal at low intensities near threshold. In summary, although substantial evidence suggests suppression during the combination of vibrotactile stimuli, relatively little work has measured psychophysical thresholds, and a corresponding computational model has yet to be developed. Importantly, quantifying the degree of tactile suppression will allow a direct comparison with vision and audition. This will enable us to determine whether differences across modalities arise from distinct mechanisms or share computational processes.

Here, we combine psychophysical measurements (Experiment 1) with EEG recordings (Experiment 2) to investigate human perception of flutter stimulation and to fit a computational model describing the combination and suppression of flutter signals. In Experiment 1, we measure how thresholds vary across different conditions and baseline levels using a two-interval forced-choice (2IFC) task. Our aim is to investigate two processes: the summation effect (by doubling the number of stimulated digits), and the masking effect (by introducing an additional mask stimulus) (see *Psychophysical procedures*). Thresholds decreased by approximately 1 dB when the number of stimulated digits was doubled, a finding consistent with probability summation rather than physiological summation. In psychophysical tasks, probability summation refers to improved detection performance resulting from multiple independent detection opportunities at the decision level [17], whereas physiological summation reflects the neural integration of sensory signals across populations of neurons. Our computational modelling indicated that this effect could potentially be explained without requiring suppression between digits. This is because psychophysical thresholds are determined primarily by the most sensitive subset of neurons relevant to the task, and suppression does not necessarily impact this 2IFC task. In comparison, EEG recordings reflect the overall activity of the entire neuronal population, where suppression between neurons is an important characteristic of sensory perception. Therefore, in our EEG Experiment, we tested these neural mechanisms directly by measuring steady-state somatosensory evoked potentials (SSSEPs). We examined summation by stimulating adjacent digits at the same frequency, and suppression by introducing a second frequency simultaneously (see *EEG procedures*). These different frequencies allowed us to isolate suppressive interactions between digits without the complicating factor of summation. The EEG modelling results indicated an inhibitory weight of suppression of 𝜔 ∼ 0.5, intermediate between the weights observed for vision (𝜔 ∼ 1) and audition (𝜔 ∼ 0).

## Materials and methods

### Participants

Eight adult subjects (7 females, aged 23–42 years) participated in the psychophysics experiment, and thirty-one adult subjects (24 females, aged 18–42 years) participated in the EEG experiment. In psychophysical studies, it is common to include a relatively small number of participants, because each individual completes a large number of trials (around 7555 trials per participant in the present study). The extensive within-participant data substantially reduces measurement noise and provides adequate statistical power to detect effects between conditions or stimulus levels, even with a modest sample size [18]. All participants self-reported as healthy, with no diagnosed neurological disorders, and no history of exposure to severe hand-transmitted vibration. Both experiments were approved by the Ethics Committee of the Department of Psychology at the University of York (application IDs 2277 and 2303). Written informed consent was obtained from all participants prior to conducting the experiments. Participants were recruited between 14 November 2023 and 16 April 2024.

### Apparatus & stimuli

Flutter stimuli were generated by a specially constructed board with ten fixed solenoids (‘tactor’ devices, from Dancer Design Ltd., Yorkshire, UK) controlled by a computer (Fig 1b). Each solenoid could be independently driven by a pair of 6-channel USB sound cards to vibrate each finger. The outputs of the sound cards were amplified by a 10-channel linear amplifier, which produced a maximum output modulation of ±7.5V, for a maximum stimulation amplitude of ±0.375N. All the stimuli were generated and presented using MATLAB and Psychtoolbox 3 [19,20]. Any sounds produced by the flutter stimuli were rendered inaudible by playing a 440 Hz tone (approximately 70 dB(A)) on the remaining two sound card outputs, which was delivered to participants over a pair of headphones.

EEG signals were recorded using a 64-channel electrode cap and an ANT Neuroscan (ANT Neuro, Netherlands) amplifier sampling at 1kHz. Electrodes were arranged according to the 10–20 system, and impedances were kept below 5𝑘Ω. Digital triggers were sent to the EEG amplifier using a USB TTL module (Black Box Toolkit Ltd., UK), signifying the start of the trial. The whole head average was used as a reference for the EEG data, and the ground electrode was located at position 𝐴𝐹𝑧.

### Psychophysical procedures

In Experiment 1, a two-interval forced-choice (2IFC) task was used to measure detection and discrimination thresholds. During the experiment, participants were instructed to place their ten digits on the corresponding solenoids, and a series of 26 Hz flutter stimuli were delivered to their hands. The digits on each hand were coded 1–5 from the thumb to the little finger in that order. The digits 1, 3 and 5 on the left hand and 2 and 4 on the right hand are denoted “Set A”, and the digits 2 and 4 on the left hand, and 1, 3 and 5 on the right hand are denoted “Set B” (illustrated in Fig 1c). Each trial consisted of two 500 ms intervals: one containing the baseline stimulus (equivalent to a pedestal stimulus in studies of visual contrast discrimination) and the other containing the baseline stimulus plus a target increment, separated by a 400 ms interstimulus interval. The next trial began 200 ms after the participant responded. To mask any sound produced by the solenoids and indicate the stimulus intervals, a 440 Hz beep sound was delivered through headphones simultaneously with the flutter stimuli. The order of the two intervals was randomised, and participants were required to determine which interval contained the target increment by pressing a foot pedal. Feedback was provided by a coloured square on the computer screen, with green indicating a correct response and red indicating an incorrect one. The amplitude of the target increment was determined using a pair of 3-down-1-up staircases, with a step size of 3 dB (where dB units are defined as 20 × 𝑙𝑜𝑔10(100 × *I*), and *I* is the stimulus intensity expressed as a proportion of the maximum system output), aiming to distribute trials around the detection threshold at 75% correct. Threshold measurement was terminated after either 70 trials or 12 reversals, whichever occurred first.

The baseline and target stimuli were presented under four conditions (illustrated in Fig 1d) and at eight baseline intensity levels (0, 0.5, 1, 2, 4, 8, 16, 32%), corresponding to force amplitudes between 0 and 0.12 N (maximum stimulation amplitude: 0.375 N). In the “pentadactyl” condition (meaning five-fingered in Greek), the baseline and target stimuli vibrated only “Set A” (analogous to the stimulation of one eye or one ear). In the “dekadactyl” (ten-fingered) condition, the baseline and target stimuli vibrated both “Set A” and “Set B” (analogous to the stimulation of both eyes or both ears). Comparing the “pentadactyl” and “dekadactyl” conditions reveals the summation effect of doubling the number of inputs. In the “dichodactyl” condition (named for consistency with dichoptic conditions in vision experiments, where different stimuli are shown to the two eyes), the baseline stimulus vibrated “Set A” and the target stimulus vibrated “Set B”, permitting the measurement of masking effects across digits. The “half-dekadactyl” condition involved vibrating all ten digits for the baseline stimulus, while only “Set A” vibrated for the target stimulus, enabling measurement of summation (by comparison with the dekadactyl condition) while controlling the number of digits receiving the baseline stimulus (see also the half-binocular condition of ref. [13]). In all conditions, the flutter stimulation frequency for both baseline and target stimuli was 26 Hz. “Set A” and “Set B” were counterbalanced across trials. Each baseline intensity level was tested in a single block lasting approximately 15 minutes and repeated three times. The entire experiment took around 7 hours per participant, resulting in a total of 60438 trials (7555 per participant on average), and was completed over multiple days.

### EEG procedures

After EEG cap set-up, participants in Experiment 2 were exposed to a series of flutter stimulation under six different conditions (illustrated in Fig 1e) at five intensity levels (4, 8, 16, 32, 64%), corresponding to force amplitudes between 0.015 and 0.24 N. Steady-state somatosensory evoked potential (SSSEP) signals were recorded throughout the experiment. During each trial, participants received an 11-s flutter stimulation with a 3-s interstimulus interval, and were required to keep their hands still until designated break periods. The “Set A” and “Set B” allocations were the same as those used in the psychophysical experiment (see Fig 1c) and were counterbalanced across trials. The order of conditions was randomised, and each condition was repeated twice for each set, resulting in a total of 120 trials. The entire experiment lasted approximately 30 minutes, split into four 7-minute blocks with rest breaks between blocks.

Two different frequencies were used: 26 Hz (F1) and 23 Hz (F2). F1 was selected as the primary stimulation frequency because SSSEP responses to hand vibration are typically maximal between approximately 20 and 40 Hz, with the greatest SSSEP responses around 26 Hz [21]. Selecting this frequency ensured the optimal signal-to-noise ratio. Using two distinct frequencies enables measurement of both the separate responses to each frequency and suppression between them, without confounding effects from summation. In the “pentadactyl” condition, F1 only vibrated “Set A”; in the “dekadactyl” condition, F1 vibrated both “Set A” and “Set B”; in the “dichodactyl” condition, F1 vibrated “Set A” while a mask at 26 Hz with an intensity level of 32% vibrated “Set B”. These three conditions contributed to the observation of summation and suppression effects at the same frequency.

In the remaining three conditions, a second frequency (F2) was introduced to investigate interactions between different frequencies. In the “cross-pentadactyl” condition, F2 vibrated “Set A”, providing a comparison with the “pentadactyl” condition and serving as a baseline for other cross-frequency conditions. In the “cross-dekadactyl” condition, F1 vibrated “Set A” and F2 vibrated “Set B”, permitting measurement of suppression effects between different frequencies. In the “cross-dichodactyl” condition, F1 vibrated “Set A” while the F2 mask (intensity level of 32%) vibrated “Set B”, again to measure suppression between digits (see Fig 1e for a diagram of all conditions).

### Data analysis

For both experiments, off-line analysis and statistical testing was performed in Python 3. For psychophysical data, the 𝑝𝑠𝑖𝑔𝑛𝑖𝑓𝑖𝑡 4 package [22] was used to estimate thresholds (at 75% correct) and slope parameters of the psychometric functions by fitting a cumulative Gaussian, parameterised as:


$$\begin{array}{c} p=\gamma +(\text{1}-\lambda -\gamma )\Phi \left(\text{C}\frac{x-m}{\omega }\right) \end{array}$$ (5)

where 𝜆 and 𝛾 define the upper and lower asymptotes (the lapse rate and baseline accuracy), 𝑥 is the stimulus intensity (in logarithmic units), 𝑚 is the threshold parameter, and ω determines the slope. (Note that 𝛾 here is unrelated to 𝛾 in Equation 6, but we retain this notation for consistency with that used in ref. [22]) Φ denotes the cumulative standard normal distribution, and 𝐶 is a constant ($C=\Phi^{-\text{1}}(\text{0}.\text{9}\text{5})-\Phi^{-\text{1}}(\text{0}.\text{0}\text{5})=\;\text{3}.\text{2897073 }$) (for details, see ref. [22]). We then converted the slope parameter to equivalent Weibull 𝛽 values using the approximation 𝛽 = 10.3/𝜎, where 𝜎 = ω/𝐶. We performed this conversion because Weibull 𝛽 is a more commonly used measure of slope, which makes for easier comparison with previous studies, and because steep slopes intuitively correspond to large 𝛽 values. Psychometric function fitting was conducted independently for each participant and condition, and we calculated geometric means across participants of the threshold and slope parameters (implemented as the arithmetic mean of logarithmic values).

For EEG data, all preprocessing was conducted using MNE-Python [23]. For each trial, the initial 1000 ms post-stimulus presentation was discarded to eliminate onset transients. The remaining 10 s were Fourier transformed and the amplitudes were averaged across repetitions and participants. After removing one outlier, data from 30 participants remained for calculating the intensity-response functions and performing statistical analysis. The amplitudes from six electrodes centred on the area of greatest response (𝐹1, 𝐹2, 𝐹𝑧, 𝐹𝐶1, 𝐹𝐶2, 𝐹𝐶𝑧) were then averaged to plot the amplitude spectra and intensity-response functions. The primary dependent variables were the Fourier amplitudes at 26 Hz and 23 Hz.

### Computational modelling

The psychophysical data were fitted using the two stage model [13], as outlined in Equations 1–4, with 7 free parameters (𝑚, 𝑆, 𝜔, 𝑝, 𝑞, 𝑍 and 𝑘). The final parameter (𝑘) determines the threshold criteria, such that the model response in the target interval must exceed that in the null interval by 𝑘 for threshold to be reached. It is proportional to additive noise in the model. We also considered a modified model, in which the summation process described by Equation 3 involved an additional exponent:


$$\begin{array}{c} sum{=(\overset{\gamma }{\underset{L}{Stage\text{1}}}+\;\overset{\gamma }{\underset{R}{Stage\text{1}}})}^{\frac{\text{1}}{\gamma }} \end{array}$$ (6)

The Minkowski exponent (𝛾) is implicitly 1 in Equation 3, but was free to vary in our second model.

To fit the EEG data, we used a simpler model described by Equations 1–3, but omitting the output nonlinearity described by Equation 4, which serves primarily to determine the shape of the dipper function in psychophysical studies, but is not required for EEG data [1]. The output was instead defined as:


$$\begin{array}{c} esp=\;R_{max}\;\times sum+k \end{array}$$ (7)

where 𝑅_𝑚𝑎𝑥_ is a scaling parameter on the output, and 𝑘 again represents additive noise. The simpler model had 5 free parameters (𝑚, 𝑆, 𝜔, 𝑅_𝑚𝑎𝑥_ and 𝑘).

We used a downhill simplex algorithm to minimise the squared error between the model and data. Fitting for each model was repeated 100 times from random starting vectors, and the parameters that gave the best numerical fit were selected. For the psychophysical data, we fitted the model to the averaged threshold data by determining the target contrast for each condition that was required to increase the model response by the criterion (𝑘).

## Results

### Summation and suppression effects on flutter thresholds

**Fig 2:**
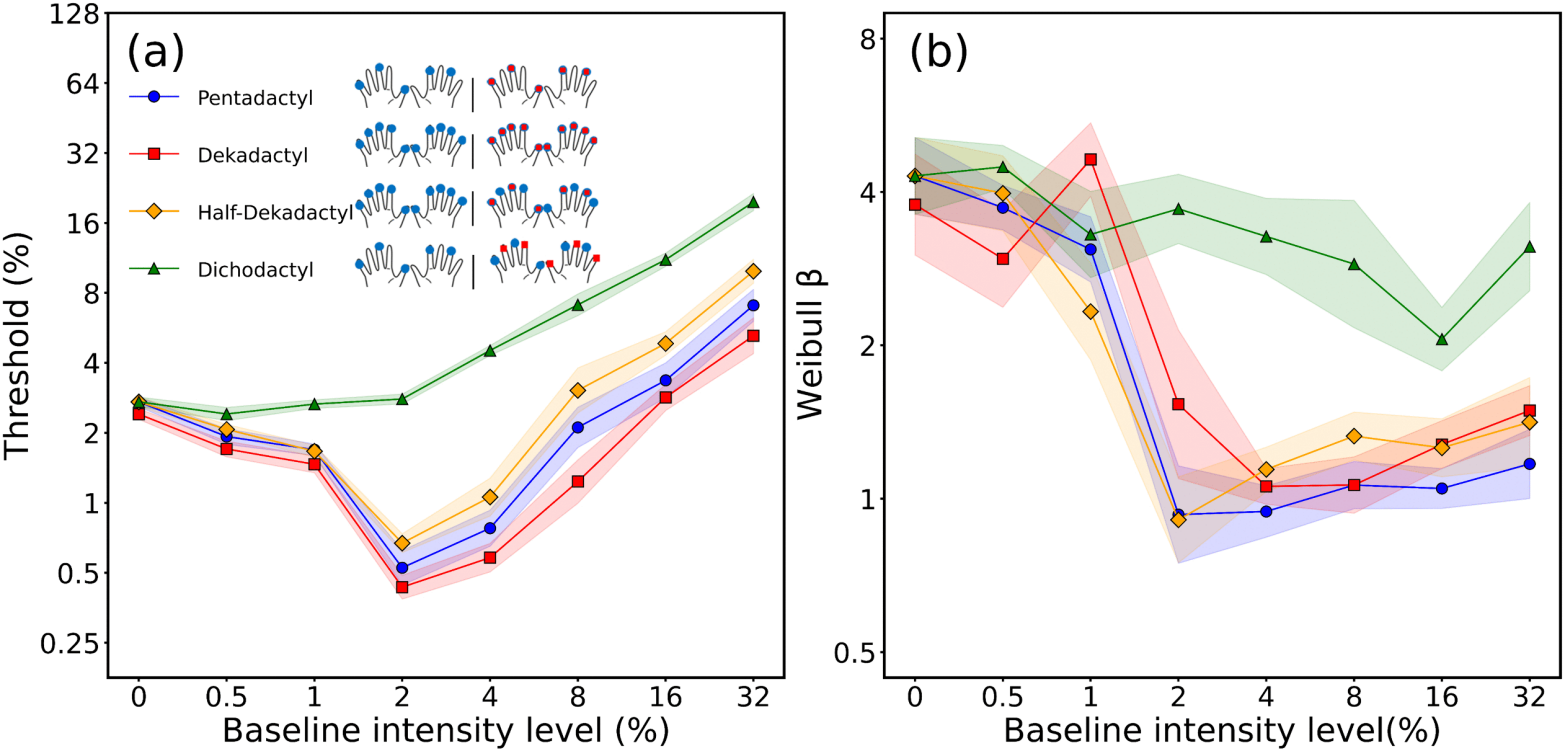
Thresholds (a) and psychometric slope values (b) from Experiment 1. Data are averaged across N = 8 participants, with shaded regions indicating ±1SE across participants.

In Experiment 1, we used a 2IFC task to measure participants’ detection and discrimination thresholds. Each trial consisted of two stimulus intervals: one containing only the baseline stimulus and the other containing the baseline plus a target increment. Participants were required to report which interval included the target stimulus (stronger flutter stimulus). Individual thresholds were measured using a 3-down-1-up staircase procedure. These data were used to fit psychometric functions, estimating thresholds at 75% correct and slope parameters via cumulative Gaussians. The average thresholds (geometric means) across eight participants for each condition and baseline intensity level (equivalent to the pedestal contrast in studies of visual contrast discrimination) are shown in Fig 2a. A 4 (condition) × 8 (baseline intensity level) repeated measures ANOVA was used to assess statistical differences between factors. We found significant main effects of condition (F_(1.66, 11.65)_ = 233.52, 𝑝 < 0.001, $\overset{\text{2}}{\underset{G}{\eta }}$ = 0.651, Greenhouse-Geisser corrected) and baseline intensity level (F_(1.45, 10.14)_ = 70.87, 𝑝 < 0.001, $\overset{\text{2}}{\underset{G}{\eta }}$ = 0.83, Greenhouse-Geisser corrected), as well as a significant interaction between the two factors (F_(21,147)_ = 22.06, 𝑝 < 0.001, $\overset{\text{2}}{\underset{G}{\eta }}$ = 0.419, Greenhouse-Geisser corrected). When plotting the thresholds against baseline intensity level, except for the dichodactyl condition (green triangles; see Fig 2a, where the baseline stimulus vibrated alternating five fingers and the target stimulus vibrated the other five fingers), the other three conditions exhibited a dipper shape (Fig 2a). Specifically, at low baseline intensity levels (0.5 to 2%), thresholds decreased with increasing baseline intensity level, indicating a facilitation effect. When the baseline intensity level exceeded 2%, thresholds increased due to a masking effect, resulting in approximately parallel ‘handles’ across the pentadactyl (blue circles; see Fig 2a, where the baseline and target stimulus vibrated alternating five fingers), dekadactyl (red squares; see Fig 2a, where both the baseline and target stimuli vibrated all ten fingers) and half-dekadactyl (orange diamonds; see Fig 2a, where the baseline stimuli vibrated all ten fingers but the target stimuli vibrated only alternating five fingers) conditions. At detection threshold (baseline intensity level = 0%), the threshold for the dekadactyl condition was around 1 dB lower than for the pentadactyl condition, with no statistically significant difference. This weak summation between digits is lower than that typically attributed to physiological summation (∼3–6 dB), suggesting a process of probability summation instead [17,24].

Above detection threshold, thresholds in the dekadactyl condition were significantly lower (*t*_(7)_ = 6.63, 𝑝 < 0.01, Bonferroni-corrected) than those in the pentadactyl condition. On average, the thresholds decreased by a factor of approximately 1.27 (2.1 dB). Additionally, thresholds in the half-dekadactyl condition, where the baseline stimulus vibrated ten digits, but the target stimulus only vibrated five digits, were higher than those in the dekadactyl condition. These findings could be interpreted as a summation effect when the target inputs were doubled. However, the psychometric function is linearised from the bottom of the dip onwards, which might increase the effects of probability summation [17]. We return to this point in the modelling section. In the dichodactyl condition, thresholds increased across all baseline intensity levels, with thresholds elevated by a factor of 7.19 (17.14 dB) at the highest baseline intensity level (32%). This result is consistent with a suppression effect when adding a mask between digits, where the target stimulus could only be detected when it approached the baseline intensity.

The slopes of the psychometric function for each condition are illustrated in Fig 2b. At detection threshold, all conditions exhibited relatively steep slopes, around 𝛽 = 4. At low baseline intensity levels (0–4%), except for in the dichodactyl condition (green triangles), the slopes decreased with increasing baseline intensity level, approaching 𝛽 = 1 at a baseline intensity level of 4%. At higher baseline intensity levels (4% to 32%), the slopes for these three conditions remained shallow, consistent with previous studies using the same paradigm in other senses [13,25]. In contrast, the slopes in the dichodactyl condition remained steep across all baseline intensity levels, with slight variations in the range 2 < 𝛽 < 5. This pattern differs from that observed in dichoptic masking in vision [13,26], where slopes were steep at detection threshold, became shallow at lower contrast levels, and then became very steep again at higher contrast levels. The very steep slopes (𝛽 ∼ 6) in dichoptic masking are attributed to mandatory physiological summation between the eyes [27]. Therefore, the approximately constant slopes observed in the dichodactyl condition suggest that mandatory physiological summation between digits may not occur here.

### Summation and suppression of neural responses

**Fig 3:**
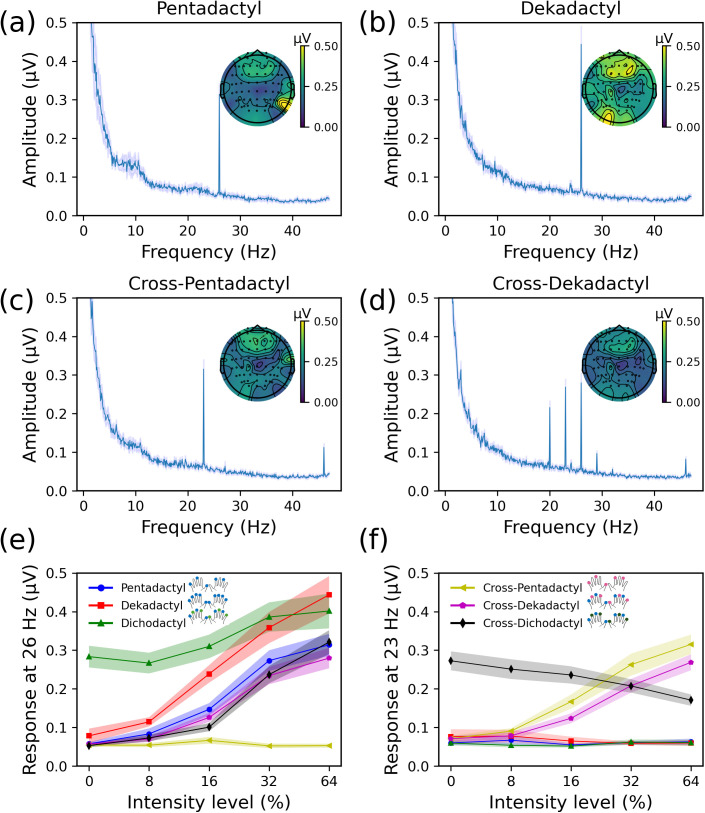
Results of the EEG experiment. Panels (a-d) show Fourier spectra and inset scalp topographies for a subset of four conditions at the highest intensity, averaged across N = 30 participants. Panels (e, f) show intensity-response functions for all conditions at 26 Hz (e) and 23 Hz (f). The frequency spectra and EEG response are averaged across 6 channels (𝐹1, 𝐹2, 𝐹𝑧, 𝐹𝐶1, 𝐹𝐶2, 𝐹𝐶𝑧). Shaded regions indicate ±1SE across participants..

To further investigate the neural mechanisms underlying these behavioural findings, we conducted an EEG experiment using the steady-state paradigm, in which participants received flutter stimuli to their fingers across six conditions and five intensity levels. The average amplitude spectra and scalp distributions for four conditions of Experiment 2 are shown in Figs 3a-d. The steady-state EEG signals were strongest at fronto-central electrodes for both frequencies, aligning with findings from previous studies involving finger stimulation in the flutter range [28,29]. Therefore, we averaged the EEG responses across six electrodes (𝐹1, 𝐹2, 𝐹𝑧, 𝐹𝐶1, 𝐹𝐶2, 𝐹𝐶𝑧) to calculate intensity-response functions at both 23 Hz and 26 Hz.

In the pentadactyl (F1 vibrated alternating five fingers; see Fig 1e) and dekadactyl (F1 vibrated all ten fingers; see Fig 1e) conditions, where a 26 Hz stimulus was used, we observed a peak at 26 Hz in both Figs 3a and b, with stronger activity at the fronto-central region in the dekadactyl condition. A similar pattern was observed in the cross-pentadactyl condition (F2 vibrated alternating five fingers; see Fig 1e), where a 23 Hz stimulus resulted in a peak at 23 Hz (Fig 3c). In the cross-dekadactyl condition (where F1 vibrated alternating five fingers and F2 vibrated the remaining fingers; see Fig 1e), when two different frequencies were presented simultaneously, peaks were observed at the original frequencies (26 Hz (F1) and 23 Hz (F2)) as well as at specific intermodulation frequencies (20 Hz = 2 × 𝐹 2 − 𝐹 1 and 29 Hz = 2 × 𝐹 1 − 𝐹 2) (Fig 3d). Additionally, the distribution of activity across the scalp was similar in the cross-dichodactyl and cross-dekadactyl conditions.

Responses increased monotonically as a function of flutter intensity for most conditions (Figs 3e, f). Two 6 (condition) × 5 (intensity) repeated measures ANOVAs were conducted on the EEG amplitudes at each frequency separately. At 26 Hz, significant main effects of condition F_(1.25, 36.28)_ = 67.32, 𝑝 < 0.001, $\overset{\text{2}}{\underset{G}{\eta }}$ = 0.309, Greenhouse-Geisser corrected), intensity level (F_(1.43, 41.55)_ = 66.6, 𝑝 < 0.001, $\overset{\text{2}}{\underset{G}{\eta }}$ = 0.288, Greenhouse-Geisser corrected), and a significant interaction effect (F_(5.60, 162.45)_ = 25.61, 𝑝 < 0.001,$\overset{\text{2}}{\underset{G}{\;\eta }}$ = 0.115) were observed. In the pentadactyl condition (blue circles), EEG amplitudes increased monotonically with increasing intensity level (Fig 3e). In the dekadactyl condition (red squares), doubling the inputs led to stronger responses. Compared to the pentadactyl condition, average response amplitudes were higher by a factor of 1.41 across all intensity levels—reflected as a multiplicative scaling of the red line. This suggests a sub-linear summation, where the combined response is greater than in the pentadactyl condition, but less than would be expected from perfect linear summation. If adjacent digit inputs were combined linearly without suppression, we would expect a doubling of response amplitude (i.e., a factor of ~2). In the dichodactyl condition (green triangles; see Fig 1e), the target stimulus was presented to Set A, while a fixed flutter stimulation at 32% of maximum intensity was presented to Set B, resulting in a high baseline response, and responses that increased with intensity level. At higher intensity levels (32–64%), the responses in the dichodactyl condition approximated those in the dekadactyl condition. In the cross-dekadactyl (pink pentagons) and cross-dichodactyl (black diamonds; Fig 1e; where F1 vibrated alternating fingers and the F2 mask, fixed at 32% of maximum intensity, vibrated the remaining fingers) conditions, two different frequencies were presented simultaneously. Instead of summing, they suppressed each other, leading to weaker responses compared to the pentadactyl condition.

At 23 Hz (Fig 3f), we also found significant main effects of condition (F_(1.39, 40.39)_ = 68.27, 𝑝 < 0.001, $\overset{\text{2}}{\underset{G}{\eta }}$ = 0.402, Greenhouse-Geisser corrected), intensity level (F_(2.55, 74.02)_ = 33.07, 𝑝 < 0.001, $\overset{\text{2}}{\underset{G}{\eta }}$ = 0.069), as well as a significant interaction effect (F_(5.47, 158.56)_ = 28.98, 𝑝 < 0.001, $\overset{\text{2}}{\underset{G}{\eta }}$ = 0.255, Greenhouse-Geisser corrected). Further evidence of suppression was observed in the cross-dekadactyl condition (pink pentagons), where simultaneous 23 Hz and 26 Hz stimuli led to reduced responses compared to the cross-pentadactyl condition (yellow triangles). In the cross-dichodactyl condition (black diamonds), we observed a strong initial response to the 23 Hz mask at 4% target intensity level. However, as the intensity of the 26 Hz target increased, the overall response decreased (F_(2.88, 83.44)_ = 14.27, 𝑝 < 0.001, $\overset{\text{2}}{\underset{G}{\eta }}$ = 0.330, Greenhouse-Geisser corrected). This negative trend suggests that the stronger the 26 Hz target, the more it suppressed the response to the 23 Hz mask [12]. The resulting pattern reflects a masking effect and inter-digit suppression, rather than summation, leading to weaker responses as intensity levels increase. As expected, the three conditions involving only a 26 Hz flutter stimulation did not show any measurable responses at 23 Hz (Fig 3f).

### Computational modelling results

**Fig 4:**
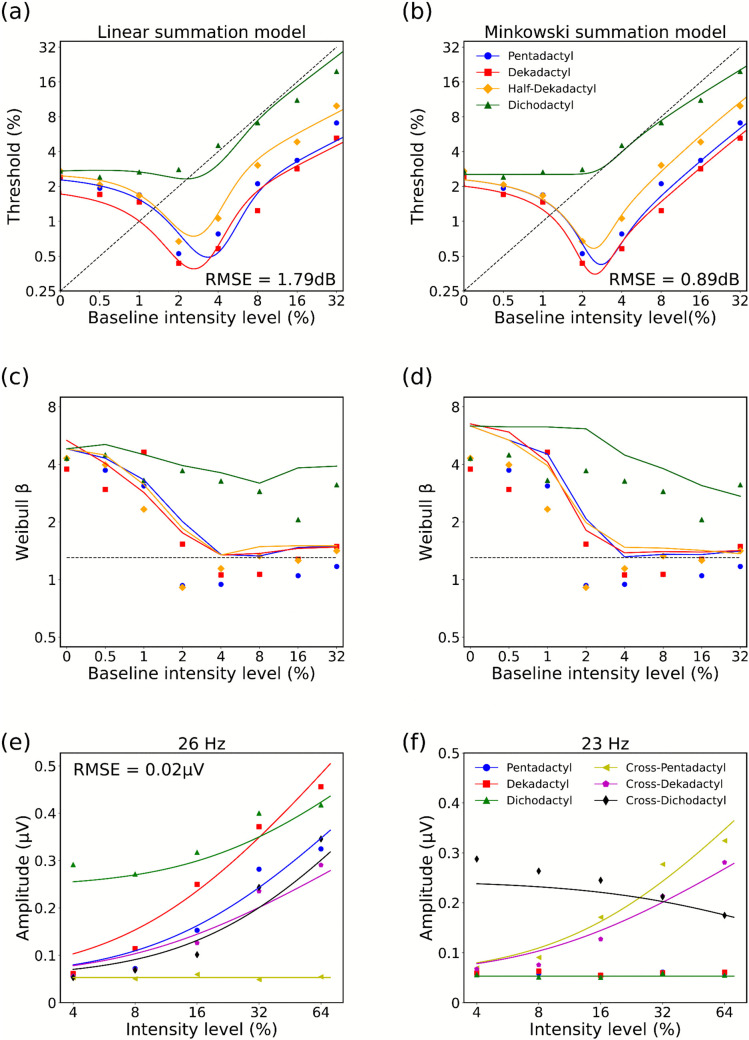
Summary of model fitting. Panel (a) shows the fit of a model in which summation is linear (following an early transducer), and panel (b) shows the fit of the Minkowski summation model. Panels (c) and (d) show the slope estimates for the Linear summation mode (c) and Minkowski summation model (d). Points are beta values estimated from the original psychophysical data at each level. Panels (e) and (f) show the best model fit to the EEG data at 26 Hz (e) and 23 Hz (f). Flat lines – yellow in (e) and red/green in (f) – correspond to unstimulated frequencies in those conditions and hence represent noise baselines.

We began by fitting the data from Experiment 1 with the model [13], which has seven free parameters, including the weight of suppression between channels (𝜔 in Equation 1 and Equation 2). Because the combination across channels is linear (following an initial transducer stage), we refer to this as the Linear summation model. The model gave a reasonable fit, as shown in Fig 4a, with an RMS error of 1.79dB (see Table 1 for parameter values). However, two systematic shortcomings are apparent. At detection threshold, the model overestimates the amount of summation (notice the red curve is below the data points for the first three baseline intensity levels). Also, suppression between channels is relatively strong (𝜔 = 0.817), but this results in the slope of the dichodactyl condition (green curve) being steeper than in the empirical data (green triangles).

**Table 1:** Summary of fitted model parameters for three computational models.

Model	*R*_max_	*p*	*q*	*m*	*S*	*Z*	*ω*	*k*	*ɤ*	RMSE
Linear summation	–	9.845	8.845	1.416	0.067	106.408	0.817	0.264	(1.0)	1.79
Minkowski summation	–	19.149	16.0	1.081	0.976	0.039	0.004	0.142	15.484	0.89
EEG model	0.053	–	–	1.263	25.955	–	0.518	0.139	–	0.02

Brackets indicate fixed values, and dashes denote parameters not included in a given model.

To address these shortcomings, we added a further parameter to the model – an exponent at the summation stage (see Equation 6), which is the defining feature of the Minkowski summation model. The Minkowski exponent determines how multiple sensory inputs are combined at the perceptual level. At high values, it serves as an approximation of probability summation, which has traditionally been modelled using a MAX operator within a 2IFC signal detection framework [17]. As the Minkowski exponent increases, the model shifts from linear integration toward a MAX-like operation, where the strongest input dominates the response. This provides a flexible way to capture both linear and nonlinear integration, depending on the value of the exponent. The Minkowski summation model provides a much more satisfactory fit (see Fig 4b), with an RMS error of 0.89dB, and no obvious systematic issues with the fit. The Minkowski exponent was estimated at 𝛾 = 15.484 — substantially greater than the implicit value of 𝛾 = 1 in the Linear summation model, and consistent with the low levels of summation at threshold in the data. Several other parameter values also differ between the models (see Table 1, more details in S1 File), most notably the weight of suppression (𝜔) reduces to near-zero. With this architecture, threshold elevation in the dichodactyl condition is caused by the MAX-like operation from the high Minkowski exponent (a true MAX operator would have an exponent of 𝛾 = ∞), meaning that explicit suppression is not required.

To predict the psychometric slopes, we used the estimated parameters from the threshold fitting for each of the two models separately. For each model, we simulated model responses across a range of target stimulus levels. For each target level and condition, we calculated d-prime (d′) as the signal difference divided by the internal noise, and obtained the predicted probability of a correct response by passing d′ divided by √2 through the cumulative normal distribution. The resulting predicted psychometric function was fitted with a cumulative Gaussian using the 𝑝𝑠𝑖𝑔𝑛𝑖𝑓𝑖𝑡 4, and the predicted slope parameters were extracted from the fitted functions. The results of psychometric slope estimates are shown in Figs 4c and 4d. Both models capture the general features of the slope data. Slopes start steep (*β* = 4) at detection threshold, and are linearised to around *β* = 1.3 at higher baseline intensities in all but the dichodactyl condition. The dichodactyl slopes remain steep (*β* > 2) across the full range of baseline intensities. We note that the additional nonlinearity in the Minkowski model does cause the slope values around detection threshold to be slightly overestimated, but the general trend is correct. Because these slope values are predicted with no additional free parameters, based on the fits at detection threshold (see also [13]), this increases our confidence in the accuracy of the models.

The absence of suppression in the Minkowski model does not necessarily mean that there is no suppression between digits — since psychophysical responses are assumed to be based on the most sensitive subset of neurons relevant to a given task, it is possible that they directly tap mechanisms that are suppression-free. Our EEG data measure responses from the whole neural population, and so we next model these results to obtain a more general estimate of suppression. The model used to fit the EEG data omits the output nonlinearity (Equation 4) and adds a scaling parameter (𝑅_𝑚𝑎𝑥_), resulting in 5 free parameters. It produced an excellent account of the results at both temporal frequencies, and across all 6 stimulus conditions (see Figs 4e and 4f), with an RMS error of 0.02𝜇V. The model correctly captures the sub-linear summation between channels (i.e., the red curve in Fig 4e is less than a factor of two greater than the blue curve), and the suppression evident in the cross-dekadactyl and cross-dichodactyl conditions. The weight of suppression (𝜔 = 0.518) was intermediate between the two dipper models, as well as being in between previously published values for vision (𝜔 = 1; see [1]) and hearing (𝜔 = 0; see [2]).

## Discussion

In this study, we present evidence detailing the processes of signal summation and suppression in vibrotactile signal combination. In the psychophysical experiment, we observed that doubling the inputs resulted in a slight reduction in detection and discrimination thresholds, indicating a weak summation effect. Additionally, adding a masking stimulus led to increased discrimination thresholds, indicating a suppression process. Furthermore, the EEG experiment demonstrated that adding inputs at the same frequency increases brain activity, whereas adding inputs at a different frequency decreases it. These findings allow us to quantify the summation and suppression effect between digits. Our computational model fitting results show that at a population level, responses to two vibrotactile inputs suppress each other, though our psychophysical data are more consistent with probability summation rather than neural/physiological summation. The weight of suppression between digits is around 𝜔 = 0.5, which is intermediate between the corresponding values observed in visual [1] and auditory [2] signal combination. Overall, our results suggest the presence of suppression in vibrotactile signal combination, with a suppression effect distinct from that observed in visual and auditory signal combination. In the remainder of this Discussion, we consider the underlying mechanisms of vibrotactile signal combination and the differences across sensory modalities.

Our results show that the detection threshold in the dekadactyl condition was ~ 1 dB lower than in the pentadactyl condition, although this difference did not reach statistical significance. Nevertheless, the trend is consistent with previous studies [15,30,31], suggesting that doubling inputs can reduce detection thresholds. However, the summation effect observed in our study was weaker than the ~ 3 dB reductions reported in those studies, which used higher-frequency stimulus (>40 Hz) and doubled the contactor size. This discrepancy may be attributed to the different stimulus frequencies used in the studies and our choice to stimulate alternating fingers rather than to vary the stimulus area. There are four types of mechanoreceptive units in the glabrous skin of the human hand, and they have been classified into four types based on their adaptation and receptive field properties: two fast-adapting types (FA I and FA Ⅱ) and two slow-adapting types (SA I and SA Ⅱ) [32]. FA I and SA I receptors are located near the skin surface and have small and well-defined receptive fields, whereas SA Ⅱ and FA Ⅱ receptors lie deeper in the skin and have large receptive fields with obscure boundaries [33]. These receptor types have distinct functional properties and respond to specific frequency ranges. For instance, FA I receptors are associated with Meissner corpuscles and are particularly sensitive to low-frequency flutter stimulation, typically in the range of approximately 5–50 Hz [34]. In contrast, FA Ⅱ receptors correspond to Pacinian corpuscles and respond preferentially to higher-frequency vibrations, roughly between 50 and 2000 Hz, with peak sensitivity ~300–500 Hz [35,36]. Pacinian corpuscles also exhibit spatial summation for vibratory stimuli. Because the stimulation frequency used in the present study (26 Hz) falls within the flutter range, the peripheral response is likely dominated by FA I receptors (Meissner corpuscles), which do not exhibit spatial summation. This may explain the relatively weak summation effect observed in our results. Additionally, unlike the study [15] that used varying contactor sizes to stimulate random areas within a test region, thereby preventing participants from knowing the exact location of stimulation, our study specifically involved the stimulation of individual digits, with participants aware of which digits were being stimulated in some conditions. This methodological difference likely contributed to the observed discrepancy between the studies.

Our EEG experiment showed a significant increase in neural responses when doubling the number of stimulated digits at the same frequency, although the increase was less than the twofold change expected if the digits were completely independent. These findings correspond with previous studies, demonstrating that brain responses to concurrent stimuli at the same frequencies are stronger than those elicited by individual stimuli, suggesting a summation effect between inputs [4,14,37]. However, the observed response is less than the anticipated summed response, generally falling between 10% and 50% of the expected value, signifying a suppression effect between inputs [4,38]. In contrast, adding a masking stimulus at a different frequency resulted in a reduction in brain activity. These results provide further evidence of suppression between digits and are consistent with previous neuroimaging studies investigating the interaction between different frequencies [39,40]. For instance, a study [40] examined the suppression effect by vibrating two fingers at the same (18 Hz) or different frequencies (18 vs. 22 Hz, 18 vs. 26 Hz). Their findings revealed that the event-related potential (ERP) and SSSEP responses to simultaneous stimulation of both fingers were significantly lower than the linear sum of individual responses, with no difference in suppression based on frequency variation. This ruled out the hypothesis that suppression is due to neuronal occlusion from overlapping cortical areas (which support adjacent areas of skin), as stronger suppression would be expected at the same frequency if this were the case [4,39]. Instead, the results support the alternative explanation of lateral inhibition, where activation of one cortical neuron suppresses neighbouring neurons’ activity [41–45]. This inhibitory mechanism has been extensively observed in both animal and human studies [3,4,14,46,47].

Another interesting finding is that we observed an intermediate level of suppression between vibrotactile stimuli compared with vision and audition. In visual perception, the forward-facing positioning of the eyes results in substantially overlapping visual fields. To merge these overlapping inputs from each eye (monocular vision) into a cohesive single percept (binocular single vision), neural signals from both eyes must exhibit strong mutual inhibition to achieve ‘ocularity invariance’, ensuring the constancy of perception through one or both eyes [48]. This is consistent with recent findings indicating that some neurons in the primary visual cortex are monocularly excitable, responding exclusively to the dominant eye, while binocular stimulation can suppress their activity [49]. In contrast, the lateral placement of the ears results in minimal overlap between auditory inputs, reducing the necessity for strong interaural inhibition to integrate these signals into a unified percept, as is required in visual processing. Instead, binaural perception benefits from the comparison and integration of these disparate inputs to localise sound sources and discern subtle differences in timing and intensity [50,51]. Although prior evidence supports the existence of binaural suppression [2,52,53], the extent of this suppression may differ depending on hemispheric laterality [11,54].

Tactile perception involves the stimulation of each of the ten fingers individually, resulting in a far more complex process of signal integration than that seen in binocular or binaural perception [55]. Stimulation delivered to different hands may be considered analogous to interactions between distinct sensory channels (e.g., the left and right eyes in vision [1]), whereas stimulation delivered to different fingers within the same hand may be more comparable to spatial interactions between nearby stimulus locations within a receptive field, such as monocular surround suppression in the visual field [12,56]. Tactile integration in the present study therefore involves both between-hand and within-hand interactions, rather than a simple two-channel interaction, which may partly explain why the observed suppression was intermediate between that reported for vision and audition.

Such interactions between fingers are thought to arise from the somatotopic organisation of the primary somatosensory cortex (S1). S1 is divided into four distinct Brodmann areas (BA 3a, 3b, 1, and 2), each responsible for mapping different parts of the body surface. Evidence suggests that receptive fields of each finger are arranged in somatotopic order in BA 3b, while in BA 1/2, they are arranged in clusters with large overlap [37,57,58]. Although SSSEPs reflect activity across the whole scalp, fMRI [59] and MEG [14,37,60] studies also support overlapping finger representations in S1. Notably, the overlap between adjacent fingers is greater than between non-adjacent ones, indicating that suppression between fingers depends on their spatial proximity. Moreover, suppression is not fixed but flexibly modulated by the perceptual goal, with stronger suppression when participants compared inputs across fingers and weaker suppression when they integrated them to form an average percept [55]. Additionally, hand posture plays a significant role in signal summation and suppression. For instance, a “CLOSE” posture (thumb and index finger positioned as if to pick something up) produces stronger suppression than an “OPEN” posture (hand fully open) [61]. In the present study, finger posture and inter-finger distance were controlled by placing the fingertips in a fixed position on solenoids, suggesting that the amount of suppression may vary depending on finger arrangements or hand posture.

In addition to interactions between fingers within the same hand, bilateral stimulation may also involve interactions between hemispheres. Somatosensory processing of unilateral tactile stimulation primarily involves activation of the contralateral S1. However, previous studies have shown that unilateral stimulation can also produce concurrent deactivation in the ipsilateral S1 and bilateral motor cortices, suggesting the presence of interhemispheric inhibition mediated through transcallosal pathways [62,63]. In the present study, stimuli were delivered to both hands across all conditions, therefore engaging somatosensory cortices in both hemispheres simultaneously. Such interhemispheric interactions may affect how tactile signals from the left and right hands are integrated. Furthermore, cortical plasticity may also influence somatosensory processing. Studies have shown that blind individuals exhibit stronger somatosensory evoked potentials compared to sighted individuals, potentially indicating an expanded S1 region [64,65]. This suggests that use-dependent cortical reorganisation may occur across different body regions, and the amount of suppression may vary depending on the body part involved.

## Conclusion

We demonstrate that vibrotactile signal combination involves both probability summation and suppression, as evidenced by psychophysical thresholds and EEG activity. A computational model further characterises this process, indicating that the strength of suppression between digits is weaker than in vision but stronger than in audition. These findings establish a foundational framework for understanding vibrotactile integration and provide a basis for future research to explore whether the weight of suppression varies across different body parts, offering deeper insights into somatosensory processing and clinical conditions in which tactile processing or body experience is affected (e.g., chronic hand pain).

## Supporting information

S1 File Supplementary Methods. (DOCX)
