# Supplementary material for: Signal combination in flutter vibration perception
Source: PLoS One. 2026 Jun 11;21(6):e0350140. doi: 10.1371/journal.pone.0350140 (PMC13258021; doi:10.1371/journal.pone.0350140)
Supplement: S1 File — (DOCX) [file pone.0350140.s001.docx]

**Supplementary Methods:**

1. **Model fitting procedure**

Model parameters were estimated by minimising the squared error between model predictions and the empirical data. Optimisation was performed using a downhill simplex algorithm. To reduce the risk of convergence to local minima, the fitting procedure was repeated 100 times from random initial parameter values, and the parameters that gave the best numerical fit were selected.

1. **Interpretation of model parameters**

**2.1 Parameter *m***

The parameter *m* determines the amount of summation at threshold in the linear summation model. The corresponding summation ratio can be expressed as $2^{1/m}$. In the present data, this ratio was approximately 1.6, which exceeds the empirically observed summation, indicating a limitation of the linear model. In the Minkowski model, *m* approaches 1, as summation is primarily governed by the Minkowski exponent (ɤ).

**2.2 Parameters *p* and *q***

The parameters *p* and *q* primarily determine the slope of the dipper handle. The fitted values in the linear summation model are comparable to those reported in previous studies (e.g., Meese et al., 2006). In the Minkowski model, *p* and *q* are larger, likely reflecting compensation for the shallower first stage transducer (controlled by *m*).

**2.3 Parameters *S, Z, k,* and *R_max_***

The parameters *S*, *Z*, and *k* control the overall sensitivity of the model (i.e., absolute detection threshold). Similarly, *R_max_* serves an analogous scaling role in the EEG model. These parameters primarily act as scaling factors to align model output with the magnitude of the empirical data and do not substantially affect the model’s behaviour.

**2.4 Parameter *ω***

The parameter *ω* determines the weight of suppression between channels, as discussed in the main manuscript. We have compared *ω* with values reported in other modalities (e.g., *ω* = 1 in vision and *ω* = 0 in audition). In the Minkowski model, we observed a small value (*ω =* 0.004), which may reflect that probability summation already provides sufficient masking in the dichodactyl condition, such that a larger value would lead to excessive masking.

**References:**

Meese TS, Georgeson MA, Baker DH. Binocular contrast vision at and above threshold. J Vis. 2006 Oct 1;6(11):7–7. doi:10.1167/6.11.7
