# Supplementary material for: Signal combination in flutter vibration perception
Source: PLoS One. 2026 Jun 11;21(6):e0350140. doi: 10.1371/journal.pone.0350140 (PMC13258021; doi:10.1371/journal.pone.0350140)
Supplement: Attachment — Submitted filename: PONE_Response letter.docx [file pone.0350140.s002.docx]

Dear Editor,

We would like to thank you and the reviewers for the careful evaluation and constructive comments on our manuscript (Manuscript ID: PONE-D-25-61332). We appreciate the opportunity to revise our work. We have carefully considered the comments and revised the manuscript to address them clearly and transparently.

**Reviewer Comments:**

**Reviewer #1:**

**1. Stimulus terminology: vibrations up to ~50 Hz are commonly called "flutter" stimuli (e.g., Romo et al., Nature 1998). Please adopt this terminology and ensure it is used consistently.**

We thank the reviewer for this suggestion. We have adopted this terminology and ensured it was used consistently throughout the text.

**2. Receptor discussion: the manuscript should better link the stimulus regime to peripheral mechanisms and to canonical receptor classes (e.g., slow‑ and fast‑adapting mechanoreceptors, e.g., Merkel, Meissner instead of Pacinian vs Non‑Pacinian I-III channels)).**

We thank the reviewer for this suggestion. We have revised the manuscript to describe the peripheral mechanisms using canonical mechanoreceptor terminology (slow‑ and fast‑adapting mechanoreceptor) rather than the Pacinian vs. non-Pacinian channel framework. We now clarify that the flutter frequency range used in this study (26 Hz) primarily engages FA I (Meissner) receptors, which are most sensitive to low-frequency vibrotactile stimulation, whereas FA II (Pacinian) receptors are preferentially activated at higher frequencies. We have revised this description in the Discussion section. See pages 19-20, lines 484-498:

“There are four types of mechanoreceptive units in the glabrous skin of the human hand, and they have been classified into four types based on their adaptation and receptive field properties: two fast-adapting types (FA I and FA Ⅱ) and two slow-adapting types (SA I and SA Ⅱ) [32]. FA I and SA I receptors are located near the skin surface and have small and well-defined receptive fields, whereas SA Ⅱ and FA Ⅱ receptors lie deeper in the skin and have large receptive fields with obscure boundaries [33]. These receptor types have distinct functional properties and respond to specific frequency ranges. For instance, FA I receptors are associated with Meissner corpuscles and are particularly sensitive to low-frequency flutter stimulation, typically in the range of approximately 5 - 50 Hz [34]. In contrast, FA Ⅱ receptors correspond to Pacinian corpuscles and respond preferentially to higher-frequency vibrations, roughly between 50 and 2000 Hz, with peak sensitivity ~300 - 500 Hz [35,36]. Pacinian corpuscles also exhibit spatial summation for vibratory stimuli. Because the stimulation frequency used in the present study (26 Hz) falls within the flutter range, the peripheral response is likely dominated by FA I receptors (Meissner corpuscles), which do not exhibit spatial summation. This may explain the relatively weak summation effect observed in our results.”

**References:**

32. Vallbo AB, Johansson RS. Properties of cutaneous mechanoreceptors in the human hand - related to touch sensation 1984.

33. Johansson RS, Vallbo ÅB. Tactile sensory coding in the glabrous skin of the human hand. Trends in Neurosciences 1983;6:27–32. https://doi.org/10.1016/0166-2236(83)90011-5.

34. Zippenfennig C, Wynands B, Milani TL. Vibration Perception Thresholds of Skin Mechanoreceptors Are Influenced by Different Contact Forces. JCM 2021;10:3083. https://doi.org/10.3390/jcm10143083.

35. Turecek J, Ginty DD. Coding of self and environment by Pacinian neurons in freely moving animals. Neuron 2024;112:3267-3277.e6. https://doi.org/10.1016/j.neuron.2024.07.008.

36. Talbot WH, Darian-Smith I, Kornhuber HH, Mountcastle VB. The sense of flutter-vibration: comparison of the human capacity with response patterns of mechanoreceptive afferents from the monkey hand. Journal of Neurophysiology 1968;31:301–34. https://doi.org/10.1152/jn.1968.31.2.301.

**3. Cross‑modal analogies: the analogy between alternating fingers and ears/eyes should be more thoroughly motivated (what aspects of the auditory/visual comparisons are being mapped onto the somatosensory case?).**

We thank the reviewer for this suggestion. We have clarified the cross-modal analogy in the Introduction and Discussion sections. Specifically, we now explain that interactions between hands may be analogous to integration across distinct sensory channels (e.g., the two eyes or ears), whereas interactions between adjacent digits resemble spatial interactions between nearby stimulus locations within a sensory receptive field.

We have now more explicitly outlined our motivation for comparing modalities in the introduction and further clarified the corresponding analogies in the Discussion.

See page 5, lines 104–106:

“Importantly, quantifying the degree of tactile suppression will allow a direct comparison with vision and audition. This will enable us to determine whether differences across modalities arise from distinct mechanisms or share computational processes.”

And pages 21-22, lines 538-544:

“Stimulation delivered to different hands may be considered analogous to interactions between distinct sensory channels (e.g., the left and right eyes in vision [1]), whereas stimulation delivered to different fingers within the same hand may be more comparable to spatial interactions between nearby stimulus locations within a receptive field, such as monocular surround suppression in the visual field [12,57]. Tactile integration in the present study therefore involves both between-hand and within-hand interactions, rather than a simple two-channel interaction, which may partly explain why the observed suppression was intermediate between that reported for vision and audition.”

Reference:

1.	Baker DH, Wade AR. Evidence for an Optimal Algorithm Underlying Signal Combination in Human Visual Cortex. Cereb Cortex. 2017 Jan;24:254–64. doi:10.1093/cercor/bhw395

12.	Busse L, Wade AR, Carandini M. Representation of Concurrent Stimuli by Population Activity in Visual Cortex. Neuron. 2009 Dec;64(6):931–42. doi:10.1016/j.neuron.2009.11.004

57.	Tsai JJ, Wade AR, Norcia AM. Dynamics of Normalization Underlying Masking in Human Visual Cortex. J Neurosci. 2012 Feb 22;32(8):2783–9. doi:10.1523/JNEUROSCI.4485-11.2012

**4. Interhemispheric effects: consider and discuss the possible role of transcallosal inhibition (i.e., interactions between hands/hemispheres) for the stimuli delivered to different hands.**

We thank the reviewer for this important suggestion. We have expanded the Discussion to consider potential interhemispheric interactions during bilateral stimulation.

We have added the following text in the revised manuscript on pages 22-23, lines 560-568:

“In addition to interactions between fingers within the same hand, bilateral stimulation may also involve interactions between hemispheres. Somatosensory processing of unilateral tactile stimulation primarily involves activation of the contralateral S1. However, previous studies have shown that unilateral stimulation can also produce concurrent deactivation in the ipsilateral S1 and bilateral motor cortices, suggesting the presence of interhemispheric inhibition mediated through transcallosal pathways [63,64]. In the present study, stimuli were delivered to both hands across all conditions, therefore engaging somatosensory cortices in both hemispheres simultaneously. Such interhemispheric interactions may affect how tactile signals from the left and right hands are integrated. Furthermore, cortical plasticity may also influence somatosensory processing.”

**References:**

63.	Hlushchuk Y, Hari R. Transient Suppression of Ipsilateral Primary Somatosensory Cortex during Tactile Finger Stimulation. J Neurosci. 2006 May 24;26(21):5819–24. doi:10.1523/JNEUROSCI.5536-05.2006

64.	Nihashi T, Naganawa S, Sato C, Kawai H, Nakamura T, Fukatsu H, et al. Contralateral and ipsilateral responses in primary somatosensory cortex following electrical median nerve stimulation—an fMRI study. Clin Neurophysiol. 2005 Apr;116(4):842–8. doi:10.1016/j.clinph.2004.10.011

**5. Model reporting: please provide fuller details of the computational model parameters (meaning of the values, fitting procedure), so readers can assess model robustness the results.**

We thank the reviewer for this suggestion. We have now provided a detailed description of the model parameters and their interpretation, along with clarification of the fitting procedure in the Supplementary Materials.

**Reviewer #2:**

**1. What was the intensity level of the auditory and vibrotactile stimulation?**

We have clarified the vibrotactile stimulation intensity levels in the Methods section. For example, in the EEG experiment, vibrotactile stimulation was delivered at 5 intensity levels (4, 8, 16, 32 and 64% of the maximum output). The maximum vibration amplitude of the stimulator was ±0.375 N; thus, the applied vibrotactile stimulation forces ranged from 0.015 to 0.24 N.

We have added the stimulation forces in the manuscript, see page 8, lines 181-183:

“The baseline and target stimuli were presented under four conditions (illustrated in Fig 1A) and at eight baseline intensity levels (0, 0.5, 1, 2, 4, 8, 16, 32%), corresponding to force amplitudes between 0 and 0.12 N (maximum vibration amplitude: 0.375 N).”

And page 9, lines 200- 202:

“After EEG cap set-up, participants in Experiment 2 were exposed to a series of vibrations under six different conditions (illustrated in Fig 1E) at five intensity levels (4, 8, 16, 32, 64%), corresponding to force amplitudes between 0.015 and 0.24 N.”

The intensity level of the auditory stimulus was approximately 70 dB(A). We have added this information to the revised manuscript, see page 7, line 153:

“Any sounds produced by the flutter stimuli were rendered inaudible by playing a 440 Hz tone (approximately 70 dB(A)) on the remaining two sound card outputs, which was delivered to participants over a pair of headphones.”

**2.Why was exactly 26 Hz used as vibration stimulus?**

We selected 26 Hz because previous work has demonstrated that somatosensory steady-state responses to hand vibration are strongest between 20 and 40 Hz, with peak amplitudes observed near 26 Hz (Snyder, 1992). Selecting this frequency ensured the optimal signal-to-noise ratio.

We have clarified this rationale as below, and also see page 9, lines 210-213 in the revised manuscript.

“F1 was selected as the primary stimulation frequency because SSSEP responses to hand vibration are typically maximal between approximately 20 and 40 Hz, with the greatest SSSEP responses around 26Hz [21]. Selecting this frequency ensured the optimal signal-to-noise ratio.”

References:

21. Snyder AZ. Steady-state vibration evoked potentials: description of technique and characterization of responses. Electroencephalogr Clin Neurophysiol Potentials Sect. 1992 May;84(3):257–68.

**3.Why was a tone used as auditory masking stimulus and not a broadband noise? A 440 Hz will have negligible masking effects on low-frequency acoustic sounds induced by the vibration stimuli, regardless of the intensity level. (Also, it must be painfully annoying to be exposed to a single continuous tone for the whole duration of the experiment.)**

We agree that broadband noise could be an alternative option, but the purpose of the auditory stimulus was to mask any potential mechanical or electronic noise from the stimulation device. This was necessary because participants could otherwise judge the relative strength of the flutter vibration by listening to the intensity of the mechanical noise. The auditory stimulus was not intended to mask low-frequency acoustic sounds produced by the flutter vibration itself.

Regarding participant comfort, the tone was presented at a moderate intensity (70 dB(A)) and only during the flutter stimulation period rather than continuously throughout the experiment. In addition, participants had multiple breaks throughout the experiment. Although a few participants commented that the sound was somewhat loud, no one reported the sound as aversive.

We hope that this version of the manuscript resolves the remaining concerns and meets the standards for publication in PLOS ONE.

Sincerely,

Shasha Wei, Alex Wade, Catherine Preston & Daniel Baker
