# Supplementary material for: Signal combination in flutter vibration perception
Source: PLoS One. 2026 Jun 11;21(6):e0350140. doi: 10.1371/journal.pone.0350140 (PMC13258021; doi:10.1371/journal.pone.0350140)
Supplement: Peer review history

# Peer review: Decision Letter 0

**DOI:** 10.1371/journal.pone.0350140.r001

**Authors:** Dimitris Voudouris

2 Mar 2026

PONE-D-25-61332Signal combination in vibration perceptionPLOS One

Dear Dr. Wei,

Thank you for submitting your manuscript to PLOS ONE. Two reviewers went through your manuscript and provided their detailed comments below. Most of them relate to methodological clarifications and revisions in the text. I believe all are doable and clearly explained, so we are looking forward to your revised manuscript.

Please submit your revised manuscript by Apr 16 2026 11:59PM. If you will need more time than this to complete your revisions, please reply to this message or contact the journal office at plosone@plos.org. When you're ready to submit your revision, log on to https://www.editorialmanager.com/pone/ and select the 'Submissions Needing Revision' folder to locate your manuscript file.

Please include the following items when submitting your revised manuscript:

- A letter that responds to each point raised by the academic editor and reviewer(s). You should upload this letter as a separate file labeled 'Response to Reviewers'.
- A marked-up copy of your manuscript that highlights changes made to the original version. You should upload this as a separate file labeled 'Revised Manuscript with Track Changes'.
- An unmarked version of your revised paper without tracked changes. You should upload this as a separate file labeled 'Manuscript'.

If you would like to make changes to your financial disclosure, please include your updated statement in your cover letter. Guidelines for resubmitting your figure files are available below the reviewer comments at the end of this letter.

If applicable, we recommend that you deposit your laboratory protocols in protocols.io to enhance the reproducibility of your results. Protocols.io assigns your protocol its own identifier (DOI) so that it can be cited independently in the future. For instructions see: https://journals.plos.org/plosone/s/submission-guidelines#loc-laboratory-protocols. Additionally, PLOS ONE offers an option for publishing peer-reviewed Lab Protocol articles, which describe protocols hosted on protocols.io. Read more information on sharing protocols at https://plos.org/protocols?utm_medium=editorial-email&utm_source=authorletters&utm_campaign=protocols.

We look forward to receiving your revised manuscript.

Kind regards,

Dimitris Voudouris

Academic Editor

PLOS One

Journal Requirements:

When submitting your revision, we need you to address these additional requirements.

1. Please ensure that your manuscript meets PLOS ONE's style requirements, including those for file naming. The PLOS ONE style templates can be found at

https://journals.plos.org/plosone/s/file?id=wjVg/PLOSOne_formatting_sample_main_body.pdf and https://journals.plos.org/plosone/s/file?id=ba62/PLOSOne_formatting_sample_title_authors_affiliations.pdf

2. Please note that PLOS One has specific guidelines on code sharing for submissions in which author-generated code underpins the findings in the manuscript. In these cases, we expect all author-generated code to be made available without restrictions upon publication of the work. Please review our guidelines at https://journals.plos.org/plosone/s/materials-and-software-sharing#loc-sharing-code and ensure that your code is shared in a way that follows best practice and facilitates reproducibility and reuse.

3. When completing the data availability statement of the submission form, you indicated that you will make your data available on acceptance. We strongly recommend all authors decide on a data sharing plan before acceptance, as the process can be lengthy and hold up publication timelines. Please note that, though access restrictions are acceptable now, your entire data will need to be made freely accessible if your manuscript is accepted for publication. This policy applies to all data except where public deposition would breach compliance with the protocol approved by your research ethics board. If you are unable to adhere to our open data policy, please kindly revise your statement to explain your reasoning and we will seek the editor's input on an exemption. Please be assured that, once you have provided your new statement, the assessment of your exemption will not hold up the peer review process.

4. We note that the grant information you provided in the ‘Funding Information’ and ‘Financial Disclosure’ sections do not match.

When you resubmit, please ensure that you provide the correct grant numbers for the awards you received for your study in the ‘Funding Information’ section.

5. Please expand the acronym “BBSRC” (as indicated in your financial disclosure) so that it states the name of your funders in full.

This information should be included in your cover letter; we will change the online submission form on your behalf.

6. If the reviewer comments include a recommendation to cite specific previously published works, please review and evaluate these publications to determine whether they are relevant and should be cited. There is no requirement to cite these works unless the editor has indicated otherwise.

7. Please review your reference list to ensure that it is complete and correct. If you have cited papers that have been retracted, please include the rationale for doing so in the manuscript text, or remove these references and replace them with relevant current references. Any changes to the reference list should be mentioned in the rebuttal letter that accompanies your revised manuscript. If you need to cite a retracted article, indicate the article’s retracted status in the References list and also include a citation and full reference for the retraction notice.

[Note: HTML markup is below. Please do not edit.]

Reviewers' comments:

Reviewer's Responses to Questions

**Comments to the Author**

1. Is the manuscript technically sound, and do the data support the conclusions?

The manuscript must describe a technically sound piece of scientific research with data that supports the conclusions. Experiments must have been conducted rigorously, with appropriate controls, replication, and sample sizes. The conclusions must be drawn appropriately based on the data presented.

Reviewer #1: Yes

Reviewer #2: Yes

**********

2. Has the statistical analysis been performed appropriately and rigorously?

Reviewer #1: Yes

Reviewer #2: Yes

**********

3. Have the authors made all data underlying the findings in their manuscript fully available?

The PLOS Data policy requires authors to make all data underlying the findings described in their manuscript fully available without restriction, with rare exception (please refer to the Data Availability Statement in the manuscript PDF file). The data should be provided as part of the manuscript or its supporting information, or deposited to a public repository. For example, in addition to summary statistics, the data points behind means, medians and variance measures should be available. If there are restrictions on publicly sharing data—e.g. participant privacy or use of data from a third party—those must be specified.

Reviewer #1: No

Reviewer #2: Yes

**********

4. Is the manuscript presented in an intelligible fashion and written in standard English?

PLOS ONE does not copyedit accepted manuscripts, so the language in submitted articles must be clear, correct, and unambiguous. Any typographical or grammatical errors should be corrected at revision, so please note any specific errors here.

Reviewer #1: Yes

Reviewer #2: Yes

**********

5. Review Comments to the Author

Please use the space provided to explain your answers to the questions above. You may also include additional comments for the author, including concerns about dual publication, research ethics, or publication ethics. (Please upload your review as an attachment if it exceeds 20,000 characters)

Reviewer #1: The study by Wei et al. "Signal combination in vibration perception" uses psychophysics and SSSEPs to show that vibrotactile inputs across fingers produce only weak behavioral summation (consistent with probability summation) and inter‑digit suppression when masked. EEG reveals about 1.4× SSSEP amplitude gain when doubling digits at the same frequency, but reduced amplitudes for different‑frequency stimulation; the results are explained by a mutual‑inhibition model with suppression strength intermediate between binocular vision and binaural hearing.

This is an interesting study, and I have only a few minor comments the authors should consider:

Stimulus terminology: vibrations up to ~50 Hz are commonly called "flutter" stimuli (e.g., Romo et al., Nature 1998). Please adopt this terminology and ensure it is used consistently.

Receptor discussion: the manuscript should better link the stimulus regime to peripheral mechanisms and to canonical receptor classes (e.g., slow‑ and fast‑adapting mechanoreceptors, e.g., Merkel, Meissner instead of Pacinian vs Non‑Pacinian I-III channels)).

Cross‑modal analogies: the analogy between alternating fingers and ears/eyes should be more thoroughly motivated (what aspects of the auditory/visual comparisons are being mapped onto the somatosensory case?).

Interhemispheric effects: consider and discuss the possible role of transcallosal inhibition (i.e., interactions between hands/hemispheres) for the stimuli delivered to different hands.

Model reporting: please provide fuller details of the computational model parameters (meaning of the values, fitting procedure), so readers can assess model robustness the results.

Reviewer #2: The present manuscript addresses the integration of vibration perception across multiple fingers. Psychophysical thresholds are measured to characterize the summation of vibrotactile stimulation together with steady state somatosensory potentials. The authors find summation effects in that stimulation at all ten fingers yield lower thresholds compared to stimulation at five fingers. Moreover, suppression from adjacent fingers is found to be present.

The paper seems to be a valuable contribution to the literature and I do not have any major concerns. My only questions refer to the description of the stimuli.

- What was the intensity level of the auditory and vibrotactile stimulation?

- Why was exactly 26 Hz used as vibration stimulus?

- Why was a tone used as auditory masking stimulus and not a broadband noise? A 440 Hz will have negligible masking effects on low-frequency acoustic sounds induced by the vibration stimuli, regardless of the intensity level. (Also, it must be painfully annoying to be exposed to a single continuous tone for the whole duration of the experiment.)

**********

6. PLOS authors have the option to publish the peer review history of their article (what does this mean?). If published, this will include your full peer review and any attached files.

If you choose “no”, your identity will remain anonymous but your review may still be made public.

**Do you want your identity to be public for this peer review?** For information about this choice, including consent withdrawal, please see our Privacy Policy.

Reviewer #1: No

Reviewer #2: No

**********

[NOTE: If reviewer comments were submitted as an attachment file, they will be attached to this email and accessible via the submission site. Please log into your account, locate the manuscript record, and check for the action link "View Attachments". If this link does not appear, there are no attachment files.]

To ensure your figures meet our technical requirements, please review our figure guidelines: https://journals.plos.org/plosone/s/figures

You may also use PLOS’s free figure tool, NAAS, to help you prepare publication quality figures: https://journals.plos.org/plosone/s/figures#loc-tools-for-figure-preparation.

NAAS will assess whether your figures meet our technical requirements by comparing each figure against our figure specifications.

# Author comment: Author response to Decision Letter 1

**DOI:** 10.1371/journal.pone.0350140.r002

30 Mar 2026

Dear Editor,

We would like to thank you and the reviewers for the careful evaluation and constructive comments on our manuscript (Manuscript ID: PONE-D-25-61332). We appreciate the opportunity to revise our work. We have carefully considered the comments and revised the manuscript to address them clearly and transparently.

Reviewer Comments:

Reviewer #1:

1. Stimulus terminology: vibrations up to ~50 Hz are commonly called "flutter" stimuli (e.g., Romo et al., Nature 1998). Please adopt this terminology and ensure it is used consistently.

We thank the reviewer for this suggestion. We have adopted this terminology and ensured it was used consistently throughout the text.

2. Receptor discussion: the manuscript should better link the stimulus regime to peripheral mechanisms and to canonical receptor classes (e.g., slow‑ and fast‑adapting mechanoreceptors, e.g., Merkel, Meissner instead of Pacinian vs Non‑Pacinian I-III channels)).

We thank the reviewer for this suggestion. We have revised the manuscript to describe the peripheral mechanisms using canonical mechanoreceptor terminology (slow‑ and fast‑adapting mechanoreceptor) rather than the Pacinian vs. non-Pacinian channel framework. We now clarify that the flutter frequency range used in this study (26 Hz) primarily engages FA I (Meissner) receptors, which are most sensitive to low-frequency vibrotactile stimulation, whereas FA II (Pacinian) receptors are preferentially activated at higher frequencies. We have revised this description in the Discussion section. See pages 19-20, lines 484-498:

“There are four types of mechanoreceptive units in the glabrous skin of the human hand, and they have been classified into four types based on their adaptation and receptive field properties: two fast-adapting types (FA I and FA Ⅱ) and two slow-adapting types (SA I and SA Ⅱ) [32]. FA I and SA I receptors are located near the skin surface and have small and well-defined receptive fields, whereas SA Ⅱ and FA Ⅱ receptors lie deeper in the skin and have large receptive fields with obscure boundaries [33]. These receptor types have distinct functional properties and respond to specific frequency ranges. For instance, FA I receptors are associated with Meissner corpuscles and are particularly sensitive to low-frequency flutter stimulation, typically in the range of approximately 5 - 50 Hz [34]. In contrast, FA Ⅱ receptors correspond to Pacinian corpuscles and respond preferentially to higher-frequency vibrations, roughly between 50 and 2000 Hz, with peak sensitivity ~300 - 500 Hz [35,36]. Pacinian corpuscles also exhibit spatial summation for vibratory stimuli. Because the stimulation frequency used in the present study (26 Hz) falls within the flutter range, the peripheral response is likely dominated by FA I receptors (Meissner corpuscles), which do not exhibit spatial summation. This may explain the relatively weak summation effect observed in our results.”

References:

32. Vallbo AB, Johansson RS. Properties of cutaneous mechanoreceptors in the human hand - related to touch sensation 1984.

33. Johansson RS, Vallbo ÅB. Tactile sensory coding in the glabrous skin of the human hand. Trends in Neurosciences 1983;6:27–32. https://doi.org/10.1016/0166-2236(83)90011-5.

34. Zippenfennig C, Wynands B, Milani TL. Vibration Perception Thresholds of Skin Mechanoreceptors Are Influenced by Different Contact Forces. JCM 2021;10:3083. https://doi.org/10.3390/jcm10143083.

35. Turecek J, Ginty DD. Coding of self and environment by Pacinian neurons in freely moving animals. Neuron 2024;112:3267-3277.e6. https://doi.org/10.1016/j.neuron.2024.07.008.

36. Talbot WH, Darian-Smith I, Kornhuber HH, Mountcastle VB. The sense of flutter-vibration: comparison of the human capacity with response patterns of mechanoreceptive afferents from the monkey hand. Journal of Neurophysiology 1968;31:301–34. https://doi.org/10.1152/jn.1968.31.2.301.

3. Cross‑modal analogies: the analogy between alternating fingers and ears/eyes should be more thoroughly motivated (what aspects of the auditory/visual comparisons are being mapped onto the somatosensory case?).

We thank the reviewer for this suggestion. We have clarified the cross-modal analogy in the Introduction and Discussion sections. Specifically, we now explain that interactions between hands may be analogous to integration across distinct sensory channels (e.g., the two eyes or ears), whereas interactions between adjacent digits resemble spatial interactions between nearby stimulus locations within a sensory receptive field.

We have now more explicitly outlined our motivation for comparing modalities in the introduction and further clarified the corresponding analogies in the Discussion.

See page 5, lines 104–106:

“Importantly, quantifying the degree of tactile suppression will allow a direct comparison with vision and audition. This will enable us to determine whether differences across modalities arise from distinct mechanisms or share computational processes.”

And pages 21-22, lines 538-544:

“Stimulation delivered to different hands may be considered analogous to interactions between distinct sensory channels (e.g., the left and right eyes in vision [1]), whereas stimulation delivered to different fingers within the same hand may be more comparable to spatial interactions between nearby stimulus locations within a receptive field, such as monocular surround suppression in the visual field [12,57]. Tactile integration in the present study therefore involves both between-hand and within-hand interactions, rather than a simple two-channel interaction, which may partly explain why the observed suppression was intermediate between that reported for vision and audition.”

Reference:

1. Baker DH, Wade AR. Evidence for an Optimal Algorithm Underlying Signal Combination in Human Visual Cortex. Cereb Cortex. 2017 Jan;24:254–64. doi:10.1093/cercor/bhw395

12. Busse L, Wade AR, Carandini M. Representation of Concurrent Stimuli by Population Activity in Visual Cortex. Neuron. 2009 Dec;64(6):931–42. doi:10.1016/j.neuron.2009.11.004

57. Tsai JJ, Wade AR, Norcia AM. Dynamics of Normalization Underlying Masking in Human Visual Cortex. J Neurosci. 2012 Feb 22;32(8):2783–9. doi:10.1523/JNEUROSCI.4485-11.2012

4. Interhemispheric effects: consider and discuss the possible role of transcallosal inhibition (i.e., interactions between hands/hemispheres) for the stimuli delivered to different hands.

We thank the reviewer for this important suggestion. We have expanded the Discussion to consider potential interhemispheric interactions during bilateral stimulation.

We have added the following text in the revised manuscript on pages 22-23, lines 560-568:

“In addition to interactions between fingers within the same hand, bilateral stimulation may also involve interactions between hemispheres. Somatosensory processing of unilateral tactile stimulation primarily involves activation of the contralateral S1. However, previous studies have shown that unilateral stimulation can also produce concurrent deactivation in the ipsilateral S1 and bilateral motor cortices, suggesting the presence of interhemispheric inhibition mediated through transcallosal pathways [63,64]. In the present study, stimuli were delivered to both hands across all conditions, therefore engaging somatosensory cortices in both hemispheres simultaneously. Such interhemispheric interactions may affect how tactile signals from the left and right hands are integrated. Furthermore, cortical plasticity may also influence somatosensory processing.”

References:

63. Hlushchuk Y, Hari R. Transient Suppression of Ipsilateral Primary Somatosensory Cortex during Tactile Finger Stimulation. J Neurosci. 2006 May 24;26(21):5819–24. doi:10.1523/JNEUROSCI.5536-05.2006

64. Nihashi T, Naganawa S, Sato C, Kawai H, Nakamura T, Fukatsu H, et al. Contralateral and ipsilateral responses in primary somatosensory cortex following electrical median nerve stimulation—an fMRI study. Clin Neurophysiol. 2005 Apr;116(4):842–8. doi:10.1016/j.clinph.2004.10.011

5. Model reporting: please provide fuller details of the computational model parameters (meaning of the values, fitting procedure), so readers can assess model robustness the results.

We thank the reviewer for this suggestion. We have now provided a detailed description of the model parameters and their interpretation, along with clarification of the fitting procedure in the Supplementary Materials.

Reviewer #2:

1. What was the intensity level of the auditory and vibrotactile stimulation?

We have clarified the vibrotactile stimulation intensity levels in the Methods section. For example, in the EEG experiment, vibrotactile stimulation was delivered at 5 intensity levels (4, 8, 16, 32 and 64% of the maximum output). The maximum vibration amplitude of the stimulator was ±0.375 N; thus, the applied vibrotactile stimulation forces ranged from 0.015 to 0.24 N.

We have added the stimulation forces in the manuscript, see page 8, lines 181-183:

“The baseline and target stimuli were presented under four conditions (illustrated in Fig 1A) and at eight baseline intensity levels (0, 0.5, 1, 2, 4, 8, 16, 32%), corresponding to force amplitudes between 0 and 0.12 N (maximum vibration amplitude: 0.375 N).”

And page 9, lines 200- 202:

“After EEG cap set-up, participants in Experiment 2 were exposed to a series of vibrations under six different conditions (illustrated in Fig 1E) at five intensity levels (4, 8, 16, 32, 64%), corresponding to force amplitudes between 0.015 and 0.24 N.”

The intensity level of the auditory stimulus was approximately 70 dB(A). We have added this information to the revised manuscript, see page 7, line 153:

“Any sounds produced by the flutter stimuli were rendered inaudible by playing a 440 Hz tone (approximately 70 dB(A)) on the remaining two sound card outputs, which was delivered to participants over a pair of headphones.”

2.Why was exactly 26 Hz used as vibration stimulus?

We selected 26 Hz because previous work has demonstrated that somatosensory steady-state responses to hand vibration are strongest between 20 and 40 Hz, with peak amplitudes observed near 26 Hz (Snyder, 1992). Selecting this frequency ensured the optimal signal-to-noise ratio.

We have clarified this rationale as below, and also see page 9, lines 210-213 in the revised manuscript.

“F1 was selected as the primary stimulation frequency because SSSEP responses to hand vibration are typically maximal between approximately 20 and 40 Hz, with the greatest SSSEP responses around 26Hz [21]. Selecting this frequency ensured the optimal signal-to-noise ratio.”

References:

21. Snyder AZ. Steady-state vibration evoked potentials: description of technique and characterization of responses. Electroencephalogr Clin Neurophysiol Potentials Sect. 1992 May;84(3):257–68.

3.Why was a tone used as auditory masking stimulus and not a broadband noise? A 440 Hz will have negligible masking effects on low-frequency acoustic sounds induced by the vibration stimuli, regardless of the intensity level. (Also, it must be painfully annoying to be exposed to a single continuous tone for the whole duration of the experiment.)

We agree that broadband noise could be an alternative option, but the purpose of the auditory stimulus was to mask any potential mechanical or electronic noise from the stimulation device. This was necessary because participants could otherwise judge the relative strength of the flutter vibration by listening to the intensity of the mechanical noise. The auditory stimulus was not intended to mask low-frequency acoustic sounds produced by the flutter vibration itself.

Regarding participant comfort, the tone was presented at a moderate intensity (70 dB(A)) and only during the flutter stimulation period rather than continuously throughout the experiment. In addition, participants had multiple breaks throughout the experiment. Although a few participants commented that the sound was somewhat loud, no one reported the sound as aversive.

We hope that this version of the manuscript resolves the remaining concerns and meets the standards for publication in PLOS ONE.

Sincerely,

Shasha Wei, Alex Wade, Catherine Preston & Daniel Baker

Attachment Submitted filename: PONE_Response letter.docx

# Peer review: Decision Letter 1

**DOI:** 10.1371/journal.pone.0350140.r003

**Authors:** Dimitris Voudouris

10 May 2026

Signal combination in flutter vibration perception

PONE-D-25-61332R1

Dear Dr. Wei,

We’re pleased to inform you that your manuscript has been judged scientifically suitable for publication and will be formally accepted for publication once it meets all outstanding technical requirements.

Within one week, you’ll receive an e-mail detailing the required amendments. When these have been addressed, you’ll receive a formal acceptance letter and your manuscript will be scheduled for publication.

An invoice will be generated when your article is formally accepted. Please note, if your institution has a publishing partnership with PLOS and your article meets the relevant criteria, all or part of your publication costs will be covered. Please make sure your user information is up-to-date by logging into Editorial Manager at Editorial Manager® and clicking the ‘Update My Information' link at the top of the page. For questions related to billing, please contact billing support.

If your institution or institutions have a press office, please notify them about your upcoming paper to help maximize its impact. If they’ll be preparing press materials, please inform our press team as soon as possible -- no later than 48 hours after receiving the formal acceptance. Your manuscript will remain under strict press embargo until 2 pm Eastern Time on the date of publication. For more information, please contact onepress@plos.org.

Kind regards,

Dimitris Voudouris

Academic Editor

PLOS One

Additional Editor Comments (optional):

Reviewers' comments:

Reviewer's Responses to Questions

**Comments to the Author**

1. If the authors have adequately addressed your comments raised in a previous round of review and you feel that this manuscript is now acceptable for publication, you may indicate that here to bypass the “Comments to the Author” section, enter your conflict of interest statement in the “Confidential to Editor” section, and submit your "Accept" recommendation.

Reviewer #2: (No Response)

**********

2. Is the manuscript technically sound, and do the data support the conclusions?

The manuscript must describe a technically sound piece of scientific research with data that supports the conclusions. Experiments must have been conducted rigorously, with appropriate controls, replication, and sample sizes. The conclusions must be drawn appropriately based on the data presented.

Reviewer #2: (No Response)

**********

3. Has the statistical analysis been performed appropriately and rigorously?

Reviewer #2: (No Response)

**********

4. Have the authors made all data underlying the findings in their manuscript fully available?

The PLOS Data policy requires authors to make all data underlying the findings described in their manuscript fully available without restriction, with rare exception (please refer to the Data Availability Statement in the manuscript PDF file). The data should be provided as part of the manuscript or its supporting information, or deposited to a public repository. For example, in addition to summary statistics, the data points behind means, medians and variance measures should be available. If there are restrictions on publicly sharing data—e.g. participant privacy or use of data from a third party—those must be specified.

Reviewer #2: (No Response)

**********

5. Is the manuscript presented in an intelligible fashion and written in standard English?

PLOS ONE does not copyedit accepted manuscripts, so the language in submitted articles must be clear, correct, and unambiguous. Any typographical or grammatical errors should be corrected at revision, so please note any specific errors here.

Reviewer #2: (No Response)

**********

6. Review Comments to the Author

Please use the space provided to explain your answers to the questions above. You may also include additional comments for the author, including concerns about dual publication, research ethics, or publication ethics. (Please upload your review as an attachment if it exceeds 20,000 characters)

Reviewer #2: (No Response)

**********

7. PLOS authors have the option to publish the peer review history of their article (what does this mean?). If published, this will include your full peer review and any attached files.

If you choose “no”, your identity will remain anonymous but your review may still be made public.

**Do you want your identity to be public for this peer review?** For information about this choice, including consent withdrawal, please see our Privacy Policy.

Reviewer #2: No

**********

# Editor report: Acceptance letter

**DOI:** 10.1371/journal.pone.0350140.r004

**Authors:** Dimitris Voudouris

PONE-D-25-61332R1

PLOS One

Dear Dr. Wei,

I'm pleased to inform you that your manuscript has been deemed suitable for publication in PLOS One. Congratulations! Your manuscript is now being handed over to our production team.

At this stage, our production department will prepare your paper for publication. This includes ensuring the following:

* All references, tables, and figures are properly cited

* All relevant supporting information is included in the manuscript submission,

* There are no issues that prevent the paper from being properly typeset

You will receive further instructions from the production team, including instructions on how to review your proof when it is ready. Please keep in mind that we are working through a large volume of accepted articles, so please give us a few days to review your paper and let you know the next and final steps.

Lastly, if your institution or institutions have a press office, please let them know about your upcoming paper now to help maximize its impact. If they'll be preparing press materials, please inform our press team within the next 48 hours. Your manuscript will remain under strict press embargo until 2 pm Eastern Time on the date of publication. For more information, please contact onepress@plos.org.

You will receive an invoice from PLOS for your publication fee after your manuscript has reached the completed accept phase. If you receive an email requesting payment before acceptance or for any other service, this may be a phishing scheme. Learn how to identify phishing emails and protect your accounts at https://explore.plos.org/phishing.

If we can help with anything else, please email us at customercare@plos.org.

Thank you for submitting your work to PLOS ONE and supporting open access.

Kind regards,

PLOS ONE Editorial Office Staff

on behalf of

Dr. Dimitris Voudouris

Academic Editor

PLOS One
